# Relaxin Inhibits the Cardiac Myofibroblast NLRP3 Inflammasome as Part of Its Anti-Fibrotic Actions via the Angiotensin Type 2 and ATP (P2X7) Receptors

**DOI:** 10.3390/ijms23137074

**Published:** 2022-06-25

**Authors:** Felipe Tapia Cáceres, Tracey A. Gaspari, Mohammed Akhter Hossain, Chrishan S. Samuel

**Affiliations:** 1 Cardiovascular Disease Program, Monash Biomedicine Discovery Institute and Department of Pharmacology, Monash University, Melbourne, VIC 3800, Australia; felipetapia.caceres@gmail.com (F.T.C.); tracey.gaspari@monash.edu (T.A.G.); 2 Florey Institute of Neuroscience and Mental Health, The University of Melbourne, Melbourne, VIC 3010, Australia; akhter.hossain@florey.edu.au; 3 Department of Biochemistry and Molecular Biology, The University of Melbourne, Melbourne, VIC 3010, Australia

**Keywords:** cardiac fibrosis, myofibroblast, NLRP3 inflammasome, relaxin, RXFP1, AT2 receptor, P2X7 receptor

## Abstract

Chronic NLRP3 inflammasome activation can promote fibrosis through its production of interleukin (IL)-1β and IL-18. Conversely, recombinant human relaxin (RLX) can inhibit the pro-fibrotic interactions between IL-1β, IL-18 and transforming growth factor (TGF)-β1. Here, the broader extent by which RLX targeted the myofibroblast NLRP3 inflammasome to mediate its anti-fibrotic effects was elucidated. Primary human cardiac fibroblasts (HCFs), stimulated with TGF-β1 (to promote myofibroblast (HCMF) differentiation), LPS (to prime the NLRP3 inflammasome) and ATP (to activate the NLRP3 inflammasome) (T+L+A) or benzoylbenzoyl-ATP (to activate the ATP receptor; P2X7R) (T+L+Bz), co-expressed relaxin family peptide receptor-1 (RXFP1), the angiotensin II type 2 receptor (AT_2_R) and P2X7R, and underwent increased protein expression of toll-like receptor (TLR)-4, NLRP3, caspase-1, IL-1β and IL-18. Whilst RLX co-administration to HCMFs significantly prevented the T+L+A- or T+L+Bz-stimulated increase in these end points, the inhibitory effects of RLX were annulled by the pharmacological antagonism of either RXFP1, AT_2_R, P2X7R, TLR-4, reactive oxygen species (ROS) or caspase-1. The RLX-induced amelioration of left ventricular inflammation, cardiomyocyte hypertrophy and fibrosis in isoproterenol (ISO)-injured mice, was also attenuated by P2X7R antagonism. Thus, the ability of RLX to ameliorate the myofibroblast NLRP3 inflammasome as part of its anti-fibrotic effects, appeared to involve RXFP1, AT_2_R, P2X7R and the inhibition of TLR-4, ROS and caspase-1.

## 1. Introduction

Myocardial repair post-injury to the heart is a process characterized by tissue inflammation [1] in association with the release of danger-associated molecular patterns (DAMPs) from the site of injury, such as ATP [2,3]. These DAMPs activate the immune system via pattern recognition receptors (PRRs), including the ATP receptor, P2X7R [4] and toll-like receptors (TLRs) [5], to clear cell debris and promote tissue inflammation and extracellular matrix (ECM) deposition [2,6,7,8]. The P2X7R is a non-selective cation channel [4] which upon activation becomes permeable to ion exchange involving potassium (K^+^), sodium (Na^2+^) and calcium (Ca^2+^) [9,10]. This receptor is expressed by several cell types including monocytes, endothelial cells, epithelial cells and (myo)fibroblasts [4,10]. Furthermore, increasing evidence has demonstrated the pivotal role of the P2X7R in inflammation and immunity [4,9,11]. P2X7R activation has also been found to be related to cytokine release (IL-1β and IL-18), nitric oxide generation and cytotoxicity [10]. Similarly, the toll-like receptor 4 (TLR-4), also expressed in (myo)fibroblasts [12], can recognize exogenous ligands (such as LPS) and DAMPs released during myocardial injury [13]. Extensive evidence has demonstrated that TLR-4 signalling activates the transcription factor, NF-κB, to promote the upregulation and maturation of pro-IL-1β and pro-IL-18 [14,15] products of the NLRP3 inflammasome.

The NLRP3 inflammasome is a tripartite structure comprised of a pattern recognition receptor, NLRP3; an apoptosis-associated speck-like protein containing a caspase recruitment domain (ASC); and caspase-1, which is responsible for the maturation of pro-IL-1β and pro-IL-18 into their active forms [16]. This complex is found in immune cells (i.e., monocytes, macrophages), as well as myofibroblasts following cardiac injury [1,14,17,18]. The NLRP3 inflammasome recognizes diverse types of cellular distress signals (i.e., mitochondrial damage, ion flux and reactive oxygen species (ROS)) induced by DAMPs [19,20,21]. Furthermore, the NLRP3 inflammasome requires two signals for assembly: (1) a TLR-4-mediated priming signal, which leads to NLRP3 and pro-IL-1β/IL-18 up-regulation [14]; and (2) an activation signal, which can be mediated by the ATP-gated ion channel P2X7R, causing NLRP3 inflammasome oligomerization and activation [6,9,20,22]. It has gradually been recognised that the therapeutic targeting of the NLRP3 inflammasome may be an effective means of ameliorating aberrant wound-healing and inflammation-induced fibrosis progression [23].

**Figure 1:**
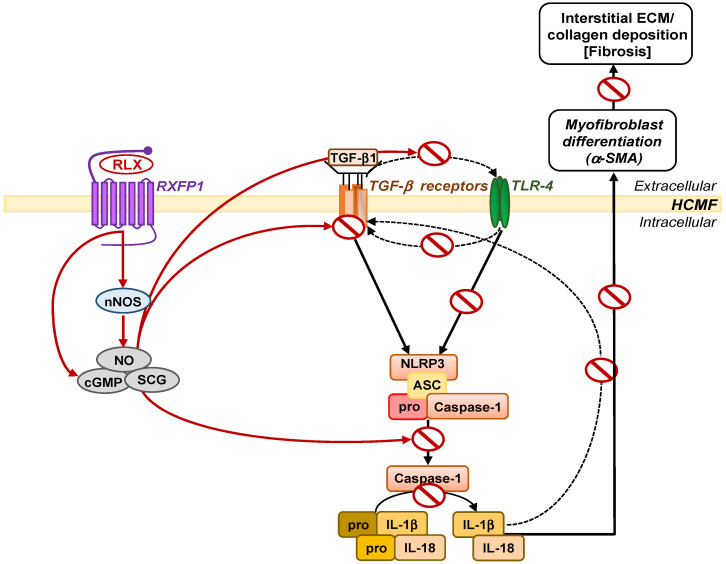
A schematic illustration of the signal transduction mechanisms by which RLX inhibits the myofibroblast NLRP3 inflammasome to mediate its anti-fibrotic actions. In RXFP1-expressing HCMFs, RLX can directly activate cGMP [29] or indirectly activate cGMP via nNOS (and a nNOS-NO-sGC-cGMP-dependent pathway [30,31]), to inhibit TLR-4 (upstream of the myofibroblast NLRP3 inflammasome) [34], as well as caspase-1 (at the level of the myofibroblast NLRP3 inflammasome) [35], as a means of disrupting the pro-fibrotic TGF-β1/IL-1β and TGF-β1/IL-18 axes. This results in the RLX-induced inhibition of TGF-β1/IL-1β and TGF-β1/IL-18-induced myofibroblast differentiation and myofibroblast-mediated ECM/collagen deposition (fibrosis).

Recombinant human relaxin (RLX, also known as serelaxin) is the drug-based version of the naturally-occurring hormone, human gene-2 (H2) relaxin, and is well-known for its anti-fibrotic actions via its ability to inhibit TGF-β1 signal transduction and pro-fibrotic activity, via the relaxin family peptide receptor 1 (RXFP1) [24,25,26]. Furthermore, the anti-fibrotic actions of RLX appear to require the angiotensin II type 2 receptor (AT_2_R) [27,28], and can be mediated via the direct activation of cyclic guanosine monophosphate (cGMP) [29] or via the phosphorylation of extracellular signal-regulated kinase (pERK1/2) and a neuronal nitric oxide (NO) synthase (nNOS)-NO-soluble guanylate cyclase (sGC)-cGMP-dependent pathway to inhibit TGF-β1 signal transduction at the level of Smad2 [27,29,30,31] and Smad3 [29,32] phosphorylation. This consistently results in the RLX-induced suppression of TGF-β1-induced myofibroblast differentiation and myofibroblast-mediated ECM/collagen deposition (which forms the basis of fibrosis), and also allows RLX to indirectly signal through AT_2_R-associated protein phosphatases [33]. It was recently demonstrated that RLX was able to suppress TGF-β1-induced myofibroblast differentiation and interstitial collagen (collagen I) deposition through a RXFP1-nNOS-TLR-4-NLRP3 inflammasome-dependent mechanism in human cardiac myofibroblasts (HCMFs) and the left ventricle of mice with cardiomyopathy, leading to the amelioration of the pro-fibrotic influence of the TGF-β1/IL-1β and TGF-β1/IL-18 axes on cardiac fibrosis progression [34] (Figure 1). Separate studies also showed that RLX could directly modulate myofibroblast NLRP3 inflammasome activity at the level of caspase-1 [35], confirming that the targeting of the myofibroblast NLRP3 inflammasome could effectively impede fibrosis progression.

In the present study, we extended the above-mentioned findings to determine the broader extent by which RLX targeted the myofibroblast NLRP3 inflammasome to mediate its anti-fibrotic effects in HCMFs in vitro and in a murine model of cardiomyopathy in vivo. In particular, we investigated the extent to which (1) RXFP1, AT_2_R, P2X7R, TLR-4, ROS (which is another trigger of NLRP3 inflammasome activity) and caspase-1 were involved in the NLRP3 inflammasome-inhibitory effects of RLX in HCMFs in vitro; and (2) the P2X7R was involved in the NLRP3-inhibitory, anti-fibrotic and cardioprotective effects of RLX, in mice with cardiomyopathy in vivo.

## 2. Results

### 2.1. Determination of the Optimal Dose of Bz-ATP (Bz) and Pharmacological Inhibitors of P2X7R, TLR-4, ROS and Caspase-1 to Be Used on HCMFs In Vitro

**Figure 2:**
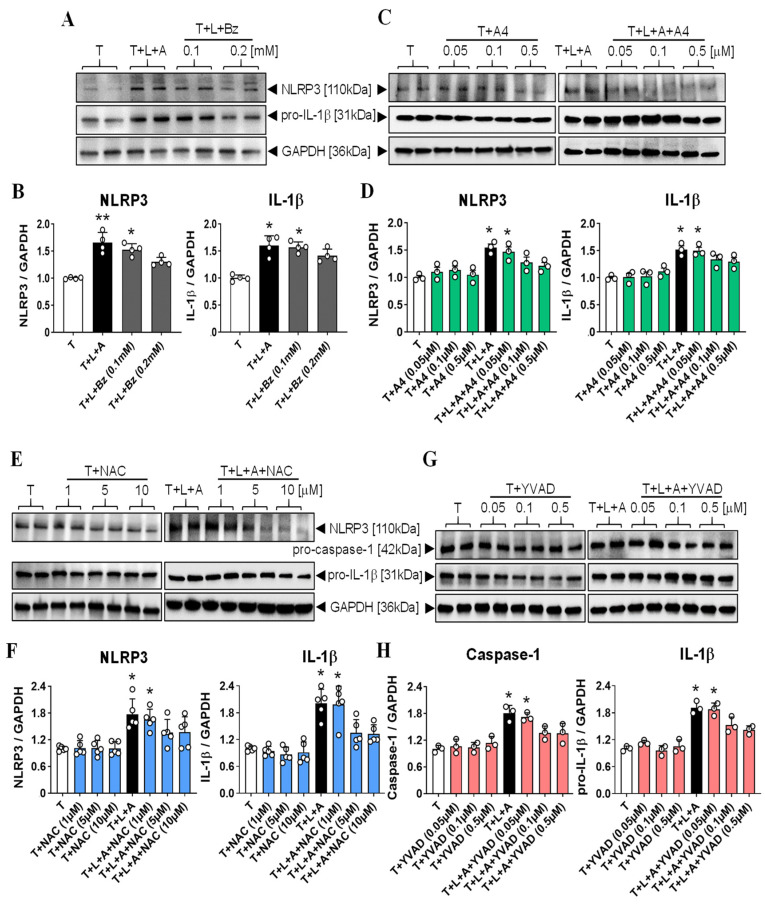
Determination of the optimal dose of T+L+Bz-ATP (Bz) that stimulated NLRP3 and pro-IL-1β to a similar extent as T+L+A (5 mM), the and optimal dose of the P2X7R inhibitor (A4), the ROS inhibitor (NAC), and the caspase-1 inhibitor (YVAD) that did not affect the T+L+A-stimulated increase in NLRP3 or pro-caspase-1 (YVAD) or pro-IL-1β expression, when administered to HCMFs alone, after 8 h. (**A**) Representative Western blots of NLRP3 and pro-IL-1β expression from T-, T+L+A (5 mM)- or T+L+Bz (0.1 or 0.2 mM)-stimulated HCMFs after 8 h in culture. (**C**,**E**,**G**) Representative Western blots of NLRP3 and pro-IL-1β expression show the effects of T- vs. T+L+A-stimulation of HCMFs treated with increasing doses of A4 (0.05–0.5 µM) (**C**), NAC (1–10 µM) (**E**), or YVAD (0.05–0.5 µM) (**G**) for 8 h. (**B**,**D**,**F**,**H**) Also shown are the mean ± SEM of each end point measured from each treatment group studied, corrected for GAPDH (house-keeping protein) loading, and expressed relative to the value in the T-stimulated group, which was expressed as 1 in each case, from *n* = 3–5 separate experiments conducted in duplicate. The individual data sets for each group are represented by the white circles within each set of bar graphs. * *p* < 0.05, ** *p* < 0.01 compared with the T alone group.

Consistent with previous findings [34], Western blot analysis of TGF-β1 (T, 5 ng/mL)+LPS (L, 100 ng/mL)+ATP (A, 5 mM)-stimulated HCMFs revealed a significant increase in NLRP3 (by ~65%, as a measure of NLRP3 inflammasome priming) and pro-IL-1β (by ~60%, as a measure of NLRP3 inflammasome activity) expression after 8 h, as compared with respective measurements obtained from T alone-stimulated cells (Figure 2A,B; both *p* < 0.001 vs. T alone). This T+L+A-stimulated increase in NLRP3 and pro-IL-1β expression was similarly maintained by T+L+Bz-ATP (Bz, the specific P2X7R agonist, at 0.1 mM) treatment of HCMFs after 8 h (which stimulated NLRP3 and pro-IL-1β levels by ~56% and ~57%, respectively, both *p* < 0.01 vs. T alone), but not by double the dose (0.2 mM) of T+L+Bz treatment over the same time period (which stimulated NLRP3 and pro-IL-1β levels by ~30% and ~40%, respectively; no different from T alone in each case) (Figure 2A,B). Based on these findings, the effects of 0.1 mM T+L+Bz were used in subsequent studies to determine whether RLX modulated the myofibroblast NLRP3 inflammasome at the level of the P2X7R.

Dose-response studies of the P2X7R inhibitor, A-438079 (A4, from 0.05–0.5 µM; Figure 2C,D) and ROS inhibitor, NAC (from 1–10 µM; Figure 2E,F), determined that 0.05 µM of A4 and 1 µM of NAC, respectively, were the optimal dose of each inhibitor that did not affect the T+L+A-stimulated increase in NLRP3 or pro-IL-1β expression on their own after 8 h. Similarly, dose-response studies of the caspase-1 inhibitor, Ac-YVAD-CHO (YVAD, from 0.05–0.5 µM; Figure 2G,H), determined that 0.05 µM was the optimal dose at which it did not affect the T+L+A-stimulated increase in pro-caspase-1 or pro-IL-1β expression, on its own.

### 2.2. RLX Inhibited Myofibroblast NLRP3 Inflammasome Priming and Activity via RXFP1, AT_2_R and P2X7R In Vitro

**Figure 3:**
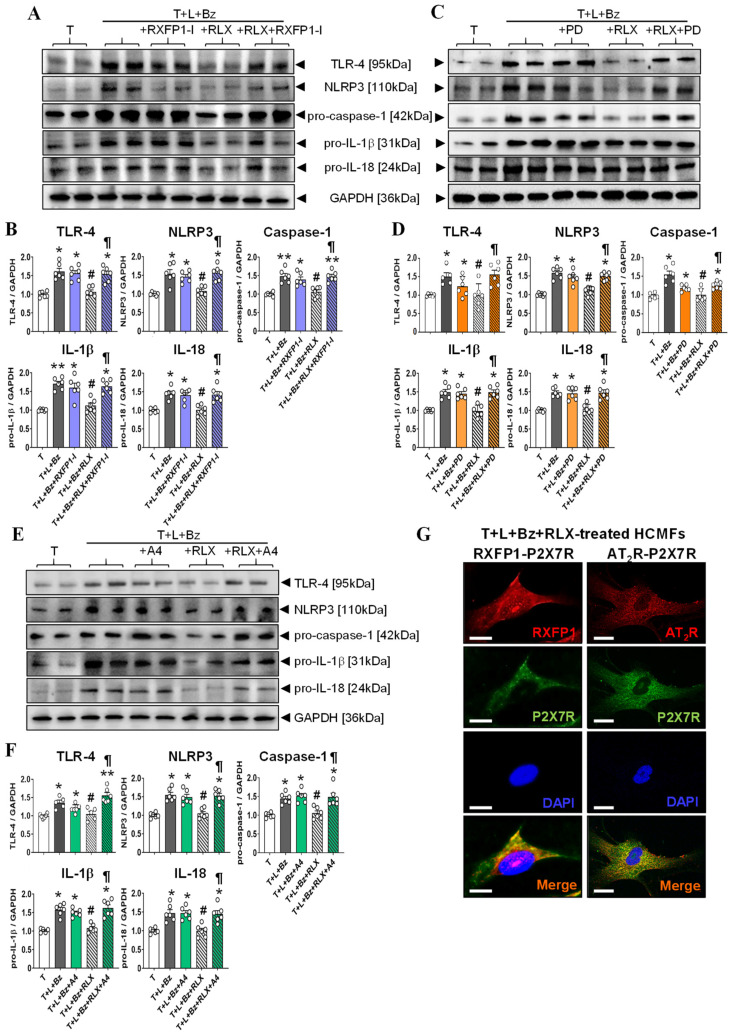
The effects of RLX ± the RXFP1 inhibitor (RXFP1-I), AT_2_R inhibitor (PD) or P2X7R inhibitor (A4) on the T+L+Bz-stimulated increase in TLR-4, NLRP3, pro-caspase-1, pro-IL-1β and pro-IL-18 levels from HCMFs. (**A**,**C**,**E**) Representative Western blots of TLR-4, NLRP3, pro-caspase-1, pro-IL-β and pro-IL-8 expression from HCMFs treated with TGF-β1 (T), T+L+Bz, T+L+Bz+RXFP1-I, T+L+Bz+RLX or T+L+Bz+RLX+RXFP1-I (**A**); T, T+L+Bz, T+L+Bz+PD, T+L+Bz+RLX or T+L+Bz+RLX+PD (**C**); or T, T+L+Bz, T+L+Bz+A4, T+L+Bz+RLX or T+L+Bz+RLX+A4 (**E**), after 8 h. (**B**,**D**,**F**) Also shown are the mean ± SEM of each end point measured from each treatment group studied, corrected for GAPDH (house-keeping protein) loading, and expressed relative to the value in the T-stimulated cell group, which was expressed as 1 in each case, from *n*=6 separate experiments conducted in duplicate. The individual data sets for each group are represented by the white circles within each set of bar graphs. (**G**) Representative immunofluorescence staining of RXFP1, AT_2_R and P2X7R, and colocalization of RXFP1 and P2X7R or AT_2_R and P2X7R in HCMFs. Scale bar = 20 μm. Nuclear staining was performed using DAPI, whilst merged images demonstrated that all three receptors were expressed by HCMFs. * *p* < 0.05, ** *p* < 0.01 compared to the T alone group; ^#^ *p* < 0.05 compared to the T+L+Bz group; ^¶^ *p* < 0.05 compared to the T+L+Bz+RLX group.

In a follow-up set of experiments, T+L+Bz (0.1 mM)-stimulated HCMFs were separately treated with or without RLX (16.8 nM) alone; or with the RXFP1-I (1 μM; Figure 3A,B), AT_2_R antagonist (PD, 0.1 μM; Figure 3C,D) or P2X7R inhibitor (A4, 0.05 μM; Figure 3E,F) alone; or the combined effects of RLX and each receptor inhibitor for 8 h. T+L+Bz stimulation of HCMFs significantly increased TLR-4, NLRP3, pro-caspase-1, pro-IL-1β and pro-IL-18 protein expression by ~45–70% after 8 h (all *p* < 0.05 vs. T alone-stimulated cells; Figure 3B,D,F). All these NLRP3 inflammasome-related end-points were markedly suppressed in T+L+Bz-stimulated HCMFs treated with RLX alone for 8 h (all by ~80–100%; all *p* < 0.05 vs. T+L+Bz treatment; all no different from T stimulation alone; Figure 3B,D,F). Whilst each receptor inhibitor on its own did not affect the T+L+Bz-stimulated increase in TLR-4, NLRP3, pro-caspase-1, pro-IL-1β and pro-IL-18 protein expression at the respective concentration administered, all three inhibitors annulled the RLX-induced suppression of the NLRP3 inflammasome-related end points measured when co-administered (all *p* < 0.05 vs. T+L+Bz+R treatment; all *p* < 0.05 vs. T stimulation alone; all no different from T+L+Bz treatment; Figure 3B,D,F).

As these findings suggested that RLX was mediating its myofibroblast NLRP3 inflammasome-inhibitory effects via RXFP1, the AT_2_R and P2X7R, colocalization staining of these receptors was performed in T+L+Bz-stimulated HCMFs (Figure 3G). As RXFP1 was previously demonstrated to undergo an interaction or crosstalk with the AT_2_R on HEK293 cells and myofibroblasts, respectively [27], it was confirmed in this study that HCMFs co-expressed the P2X7R and either RXFP1 or AT_2_R (Figure 3G), indicating that all three receptors were co-expressed by HCMFs.

### 2.3. RLX Inhibited Myofibroblast NLRP3 Inflammasome Priming and Activity via TLR-4, ROS and Caspase-1 In Vitro

**Figure 4:**
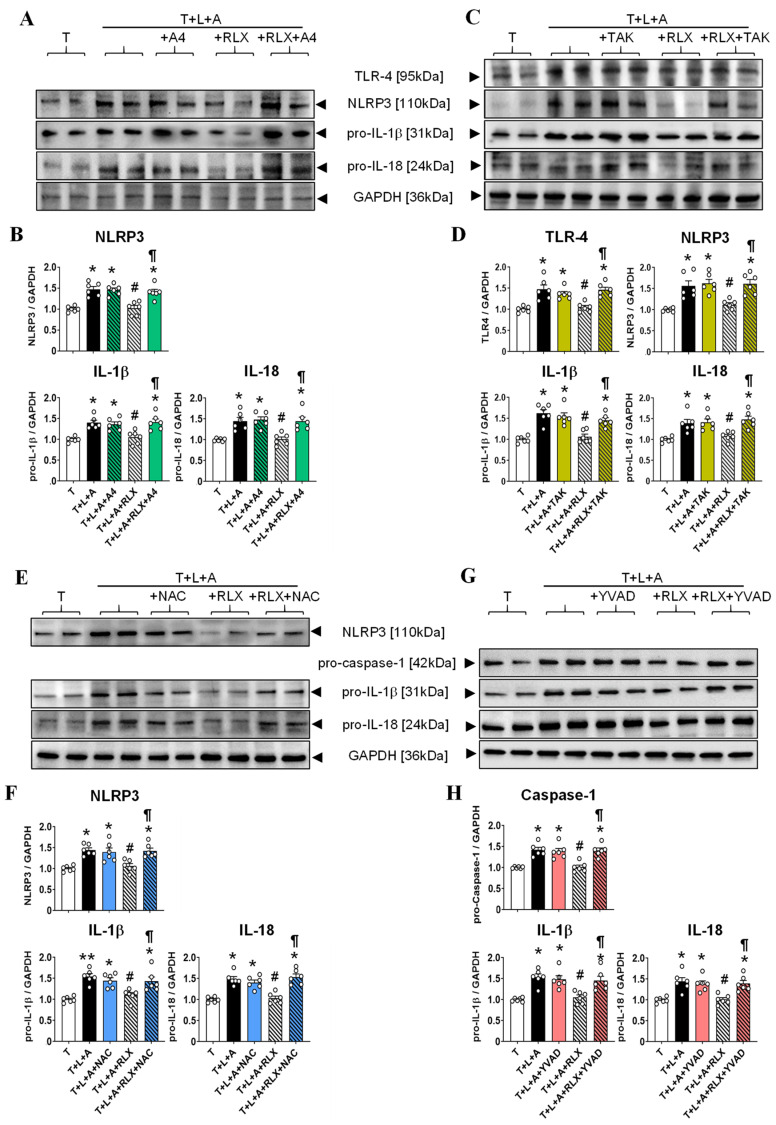
The effects of RLX ± the P2X7R inhibitor (A4), TLR-4 inhibitor (TAK), ROS inhibitor (NAC) and caspase-1 inhibitor (YVAD) on the T+L+A-stimulated increase in myofibroblast NLRP3 inflammasome measures. (**A**,**C**,**E**,**G**) Representative Western blots of TLR-4, NLRP3 (or pro-caspase-1 for the studies involving YVAD), pro-IL-β and pro-IL-8 expression from HCMFs treated with TGF-β1 (T), T+L+A, T+L+A+A4, T+L+A+RLX or T+L+A+RLX +A4 (**A**); T, T+L+A, T+L+A+TAK, T+L+A+RLX or T+L+A+RLX +TAK (**C**); T, T+L+A, T+L+A+NAC, T+L+A+RLX or T+L+A+RLX+NAC (**E**); or T, T+L+A, T+L+A+YVAD, T+L+A+RLX or T+L+A+RLX+YVAD (**G**), after 8 h. (**B**,**D**,**F**,**H**) Also shown are the mean ± SEM of each end point measured from each treatment group studied, corrected for GAPDH (house-keeping protein) loading, and expressed relative to the value in the T-stimulated cell group, which was expressed as 1 in each case, from *n* = 6 separate experiments conducted in duplicate. The individual data sets for each group are represented by the white circles within each set of bar graphs. * *p* < 0.05, ** *p* < 0.01 compared with the T alone group; ^#^ *p* < 0.05 compared with the T+L+A group; ^¶^ *p* < 0.05 compared with the T+L+A+RLX group.

In a separate set of experiments from those described above, the effects of T+L+A on measures of the NLRP3 inflammasome from HCMFs were measured in the absence or presence of RLX (16.8 nM) alone, the P2X7R inhibitor (A4, 0.05 μM; Figure 4A,B), TLR-4 inhibitor (TAK, 0.05 µM; Figure 4C,D), ROS inhibitor (NAC, 1 μM; Figure 4E,F) or caspase-1 inhibitor (YVAD, 0.05 μM, Figure 4G,H) alone, or the combined effects of RLX and each pharmacological inhibitor for 8 h. The effects of RLX ± A4 were re-evaluated in T+L+A-stimulated HCMFs to confirm that RLX was signalling via a P2X7R-mediated mechanism, even when administered to T+L+A-stimulated cells. Similar to the effects of T+L+Bz (Figure 3), T+L+A-stimulation of HCMFs significantly increased TLR-4, NLRP3, pro-caspase-1 (in studies involving YVAD), pro-IL-1β and pro-IL-18 protein expression by ~40–62% after 8 h (all *p* < 0.05 vs. T alone-stimulated cells; Figure 4B,D,F,H). All these measures of NLRP3 inflammasome priming and activity were ameliorated by RLX treatment of HCMFs (all by ~80–100%; all *p* < 0.05 vs. T+L+A treatment; all no different from T stimulation alone; Figure 4B,D,F) after 8 h. However, the RLX-induced suppression of these various end-points were all annulled by the co-administration of A4 (Figure 4B), TAK (Figure 4D), NAC (Figure 4F) or YVAD (Figure 4H), whereas neither of the pharmacological inhibitors evaluated affected the T+L+A-stimulated increase in TLR-4, NLRP3, pro-caspase-1 (in studies involving YVAD), pro-IL-1β and pro-IL-18 protein expression on their own, at the concentrations tested. These findings indicated that RLX was able to inhibit the myofibroblast NLRP3 inflammasome at the level of TLR-4, ROS and caspase-1, downstream of RXFP1, the AT_2_R and P2X7R.

### 2.4. Neither Repeated ISO Administration nor Any of the Treatments Evaluated Affected Blood Pressure in the Murine Model of Cardiomyopathy In Vivo

Systolic blood pressure (SBP) was evaluated in saline- vs. ISO-injected mice, and in ISO-injured mice subjected to RLX or A-438079 (A4) treatment alone or both treatments combined (at days 0 (Basal SBP), 7 and 14 (End SBP) in each case). ISO-injected male 129_sv/ev_ mice maintained SBP measurements of ~115–120 mmHg over the 14-day experimental period (Table 1), which was similar to that measured from their saline-treated counterparts. The administration of RLX and/or A4 treatment of ISO-injured mice (from days 7–14 post-injury) did not significantly affect SBP levels compared to the measurements for their saline-injected counterparts (Table 1). Consistent with previous studies [34,36,37], these findings confirmed that ISO induced fibrotic cardiomyopathy in mice, in the absence of any marked changes in SBP. Furthermore, these findings confirmed that the regulatory effects of RLX and/or A4 on measures of LV inflammation, remodelling and fibrosis were mediated independently of SBP regulation in the model studied.

### 2.5. Neither Repeated ISO Administration nor Any of the Treatments Evaluated Affected Heart-Weight or LV-Weight-to-BW Ratio in the Murine Model of Cardiomyopathy In Vivo

Repeated ISO administration to male 129_sv/ev_ mice did not significantly affect their body weight (BW), heart weight (HW), LV weight (LVW), HW:BW ratio or LVW:BW ratio at 14 days post-injury (Table 1), compared to respective measurements obtained from saline-treated controls. Although A4 (34.2 mg/kg/day) treatment alone significantly increased LVW (by ~21% after 7 days of treatment) over corresponding measurements from saline-injected control mice at 14 days post-injury (*p* < 0.05 vs. saline group), there was no significant difference in BW, HW:BW ratio or LVW:BW ratio between either of the five groups evaluated in this study, at 14 days post-injury (Table 1).

### 2.6. RLX Attenuated Several Measures of LV Inflammation in the ISO-Induced Murine Model of Cardiomyopathy In Vivo, via a P2X7R-Mediated Mechanism

**Figure 5:**
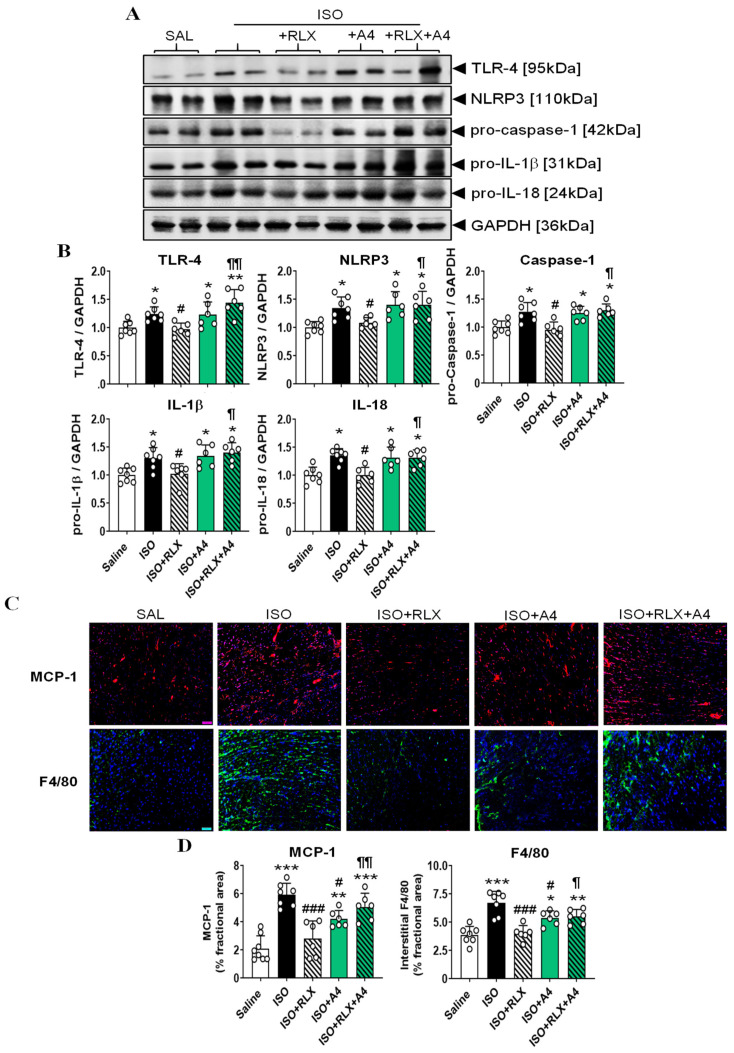
The effects of ISO as well as RLX and/or A4 treatment on measures of LV inflammation in mice. (**A**) Representative Western blots of TLR-4, NLRP3, pro-caspase-1, pro-IL-1β and pro-IL-18 expression levels from the LV of saline (SAL) or ISO-injected mice at day 14, and from ISO-injured mice that were treated with RLX (R), A4 or R+A4 from 7–14 days post-injury. (**B**) Also shown are the mean ± SEM of each end point measured from each treatment group studied, corrected for GAPDH (house-keeping protein) loading, and expressed relative to the value in the SAL-injected control group, which was expressed as 1 in each case, from *n* = 6–7 mice per group. (**C**) Representative immunofluorescent-stained images of MCP-1 (red staining) and F4/80 (green staining), with DAPI (blue) co-staining of cell nuclei, show the extent of MCP-1 and F4/80-stained macrophage infiltration within the LV of SAL- or ISO-injected mice at day 14, as well as from ISO-injured mice that were treated with RLX, A4 or RLX+A4 from days 7–14 post-injury. Scale bar = 50 μm. (**D**) Additionally shown is the mean ± SEM% fractional area of MCP-1 or F4/80 staining, from *n* = 6 non-overlapping fields per section and *n* = 6–7 mice per group. The individual data sets for each group are represented by the white circles within each set of bar graphs. * *p* < 0.05, ** *p* < 0.01, *** *p* < 0.001 compared to the SAL group; ^#^ *p* < 0.05, ^###^ *p* < 0.001 compared to the ISO group; ^¶^ *p* < 0.05, ^¶¶^ *p* < 0.01 compared to the ISO+RLX-treated group.

Repeated ISO injections to male 129_sv/ev_ mice resulted in significantly increased TLR-4, NLRP3, pro-caspase-1, pro-IL-1β, pro-IL-18 expression (by ~25–35%; Figure 5A,B), as well as MCP-1 (which plays an essential role in recruiting monocytes to the site of injury [38], by ~1.7-fold) and F4/80^+^ macrophages (a marker of macrophage infiltration [39], by ~73%; Figure 5C,D) within the LV after 14 days of injury (all *p* < 0.05 vs. respective measurements from saline-injected mice). These findings confirmed that ISO-injured mice underwent significantly increased LV inflammation at the time point studied. RLX administration to ISO-injured mice annulled all these measures of LV inflammation (all *p* < 0.05 vs. respective measurements from ISO-injured mice) to levels that were no longer different from that measured in saline-injected control mice, after 7 days of treatment (Figure 5A–D). On the other hand, A4 treatment of ISO-injured mice did not affect the ISO-induced increase in LV TLR-4 or NLRP3 inflammasome expression (Figure 5A,B), but partially and significantly reduced the ISO-induced increase in LV MCP-1 and F4/80^+^ macrophage infiltration (both by ~46%; both *p* < 0.05 vs. ISO alone group; both *p* < 0.05 vs. saline group; Figure 5C,D) at the dose administered after the 7-day treatment period. Strikingly, the RLX-induced annulment of all ISO-induced LV inflammatory end points measured was antagonized when it was co-administered with A4, after 7 days of treatment (all *p* < 0.05 vs ISO+RLX-treatment alone; all no different from ISO alone; Figure 5A–D).

### 2.7. RLX Attenuated LV Cardiomyocyte Hypertrophy and Measures of LV Fibrosis in the ISO-Induced Murine Model of Cardiomyopathy In Vivo, via a P2X7R-Mediated Mechanism

**Figure 6:**
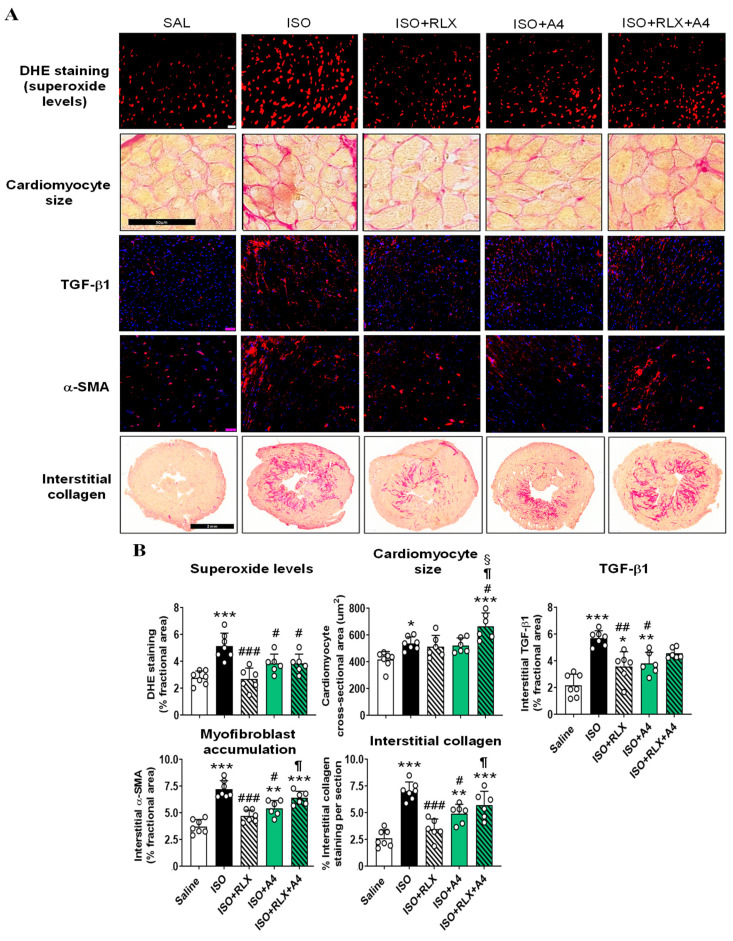
The effects of ISO as well as RLX and/or A4 treatment on measures of LV oxidative stress, cardiomyocyte hypertrophy and fibrosis. (**A**) Representative immunofluorescent-stained images of LV DHE-stained superoxide levels (red staining), cardiomyocyte size and interstitial TGF-β1 (red staining, counterstained with DAPI (blue) staining of cell nuclei) α-SMA (red staining, counterstained with DAPI (blue) staining of cell nuclei) and interstitial collagen deposition (red-staining) from saline (SAL)- or ISO-injected mice at day 14, and from ISO-injured mice that were treated with RLX (R), A4 or R+A4 from days 7–14 post-injury. Scale bar = 50 μm or 2 mm for the picrosirius red-stained LV sections. (**B**) Also shown is the mean ± SEM % fractional area of each of the end points measured, from *n* = 6 non-overlapping fields per section or ≥100 cardiomyocytes per section and *n* = 6–7 mice per group. The individual data sets for each group are represented by the white circles within each set of bar graphs. * *p* < 0.05, ** *p* < 0.01, *** *p* < 0.001 compared to the SAL group; ^#^ *p* < 0.05, ^##^ *p* < 0.01, ^###^ *p* < 0.001 compared to the ISO group; ^¶^ *p* < 0.05 compared to the ISO+RLX-treated group; ^§^ *p* < 0.05 compared to the ISO+A4-treated group.

Repeated ISO injections to male 129_sv/ev_ mice resulted in significantly increased DHE-stained superoxide levels (by ~85%), cardiomyocyte hypertrophy (by ~30%), TGF-β1, α-SMA-stained myofibroblast accumulation and interstitial fibrosis (all by ~0.9–1.7-fold) within the LV after 14 days of injury (all *p* < 0.05 vs. respective measurements from saline-injected mice; Figure 6A,B). These findings confirmed that ISO-injured mice underwent significantly increased LV oxidative stress, cardiomyocyte hypertrophy and fibrosis at the time point studied. RLX treatment of ISO-injured mice annulled the ISO-induced increase in LV DHE staining, myofibroblast accumulation and interstitial fibrosis (all *p* < 0.01 vs. ISO alone; all no different from the respective measurements from saline-injected mice); blunted the ISO-induced increase in LV cardiomyocyte hypertrophy to levels that were no longer different from that measured in saline-injected controls; and partially but significantly reduced the ISO-induced increase in LV TGF-β1 staining (by ~60%; *p* < 0.01 vs. ISO alone group; *p* < 0.05 vs. saline group) after 7 days of administration (Figure 6A,B). Comparatively, A4 treatment of ISO-injured mice partially but significantly ameliorated the cardiomyopathy-induced increase in LV DHE, TGF-β1, α-SMA and interstitial collagen staining (all by ~48–56%; all *p* < 0.05 vs. ISO alone group; *p* < 0.05 vs. saline-injected mice for the three measures of fibrosis), and also blunted the ISO-induced increase in LV cardiomyocyte hypertrophy to levels that were no longer different from that measured in saline-injected controls, after 7 days of administration (Figure 6A,B). Notably, the co-administration of RLX and A4 (over a 7-day period) to ISO-injured mice did not significantly affect the RLX-induced annulment of LV DHE or TGF-β1 staining, but significantly antagonized the protective effects of RLX on LV cardiomyocyte hypertrophy, myofibroblast accumulation and interstitial fibrosis (all *p* < 0.05 vs. ISO+RLX-treatment; all no different from ISO alone group; Figure 6A,B). Furthermore, LV cardiomyocyte size in the RLX+A4-treated group was significantly greater than that measured in all other groups (*p* < 0.001 vs. saline group; *p* < 0.05 vs. ISO alone group; *p* < 0.05 vs. ISO+RLX treatment; *p* < 0.05 vs. ISO+A4 treatment; Figure 6A,B). These findings indicated that the inhibitory effects of RLX on LV cardiomyocyte size and fibrosis appeared to be mediated via a P2X7R-dependent mechanism.

## 3. Discussion

Robust evidence has demonstrated a critical role for the NLRP3 inflammasome in the pathophysiology of various cardiovascular diseases (CVDs) owing to its ability to secrete the pro-inflammatory cytokines IL-1β and IL-18. When continually released, these cytokines in turn promote the stimulation of myofibroblast-mediated ECM/collagen deposition via a positive feedback loop with the TGF-β1/Smad3 axis, which leads to fibrosis progression and related organ stiffening and impairment [19,21,23]. Whilst much attention has focused on the macrophage NLRP3 inflammasome [23,40], owing to the initial role of macrophages in the wound-healing-associated inflammatory response to myocardial injury, the myofibroblast NLRP3 inflammasome has increasingly been recognized for its contribution to both wound healing and aberrant wound-healing-induced fibrosis [14,23,34,35]. Additionally, several studies have shown the involvement of the P2X7R in the inflammatory response to tissue injury, owing to its pro-inflammatory properties linked to cytokine release, nitric oxide generation and cytotoxicity [9,10,11]. Potassium efflux following ATP binding to the P2X7R has been linked to NLRP3 inflammasome assembly and IL-18 and IL-1β secretion [20,41].

RLX has consistently demonstrated its anti-fibrotic actions in several pre-clinical models of CVD by suppressing the pro-fibrotic TGF-β1/Smad2 [29,30,36] and/or TGF-β1/Smad3 [29,32] axes, which in turn limits the ability of TGF-β1 to promote fibroblast-to-myofibroblast differentiation and myofibroblast-mediated ECM/collagen deposition. Continuous RLX administration to RXFP1-expressing human and rodent myofibroblasts has been shown to suppress the pro-fibrotic actions of TGF-β1 via RXFP1-pERK1/2-nNOS-NO-sGC-cGMP- [30,31] and/or Notch-1-dependent [32] signalling. Furthermore, RLX was more recently shown to reduce the pro-fibrotic interaction between TGF-β1 and the myofibroblast NLRP3 inflammasome-produced IL-1β and IL-18, via a RXFP1-nNOS-TLR-4-NLRP3 inflammasome-dependent pathway in HCMFs and ISO-injured mice [34].

**Figure 7:**
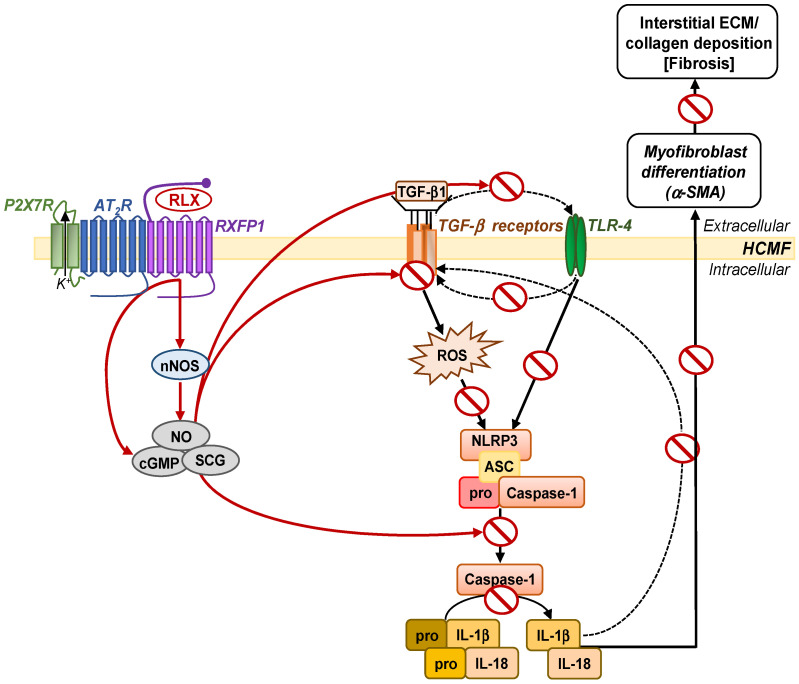
A schematic illustration summarizing the main findings of this study in relation to those of previously published findings on the RLX signal transduction of myofibroblasts [29,30,31,34,35]. It is now proposed that RLX signals through its cognate receptor, RXFP1, the AT_2_R and P2X7R on HCMFs (when all three receptors are appropriately expressed) as well as a RXFP1-nNOS-(NO-sGC-cGMP)-mediated mechanism to disrupt the induction of the myofibroblast NLRP3 inflammasome at the level of the TLR-4 (and the TLR-4/TGF-β1 axis [42]) and ROS. This study confirmed that RLX can also directly inhibit the myofibroblast NLRP3 inflammasome (at the level of caspase-1) to mediate its anti-fibrotic actions in HCMFs and the LV of ISO-injured mice. This in turn would result in the RLX-induced inhibition of the pro-fibrotic TGF-β1/IL-1β and TGF-β1/IL-18 interactions on myofibroblast differentiation and myofibroblast-mediated ECM/collagen synthesis and deposition (fibrosis).

The current study has both validate those previous findings and also provided new insights into the signal transduction mechanisms by which RLX regulated the myofibroblast NLRP3 inflammasome to inhibit the TGF-β1/IL-1β and TGF-β/IL-18 axes, as part of its anti-fibrotic actions. More specifically, the findings of this study demonstrate that the myofibroblast NLRP3 inflammasome-inhibitory effects of RLX appear to be mediated via RXFP1, AT_2_R and P2X7R, the inhibition of TLR-4 and ROS (upstream of the myofibroblast NLRP3 inflammasome), and caspase-1 (at the level of the myofibroblast NLRP3 inflammasome). This in turn likely resulted in the RLX-induced inhibition of the interaction between TGF-β1 and the myofibroblast NLRP3 inflammasome, and the impact of the myofibroblast NLRP3 inflammasome on LV inflammation and fibrosis (Figure 7). In line with the receptor crosstalk that was previously demonstrated between RXFP1 and the AT_2_R in HEK293 cells and myofibroblasts [27,33], our findings, which showed (i) the co-expression of RXFP1, AT_2_R and P2X7R in HCMFs (Figure 3G) and (ii) that the administration of RLX to T+L+Bz-stimulated HCMFs was equivalently annulled by co-administration of RXFP1, AT_2_R or P2X7R antagonism, may suggest that RLX mediates its effects via some level of crosstalk between all three receptors on HCMFs. However, further work evaluating the effects of T+L+Bz+RLX+RXFP1 inhibition/silencing on agonists or antagonists (i.e., PD or A4) that act at the AT_2_ or P2X7 receptors, or mutational (loss of function) analysis with rescue experiments, would be needed to validate the extent to which this receptor crosstalk occurs. Furthermore, although only the pro-forms of caspase-1, IL-1β and IL-18 were consistently measured throughout this study, our previous studies using TGF-β1-stimulated HCMFs [34] or human dermal myofibroblasts [35] had demonstrated that the T+L+A-stimulation of pro-caspase-1 or pro-IL-1β (or RLX-induced down-regulation of these factors) correlated with corresponding changes to the active forms of caspase-1 or IL-1β, respectively. Unexpectedly, P2X7R signalling appeared to be involved in the RLX-mediated regulation of the LV myofibroblast NLRP3 inflammasome, macrophage infiltration, cardiomyocyte hypertrophy and fibrosis, but not so in the RLX-induced regulation of LV superoxide levels or TGF-β1 expression levels per se.

These results, firstly, confirm the direct involvement of RXFP1 in the NLRP3 inflammasome-inhibitory effects of RLX. Whilst it was previously only demonstrated that the HCMFs used in this study expressed RXFP1 [34], the current study further validated that RXFP1 blockade (with the B-R13/17K H2 compound) was able to annul the myofibroblast NLRP3 inflammasome-inhibitory effects of RLX (from the level of TLR-4 to pro-IL-1β and pro-IL-18). Secondly, the results obtained align with growing evidence which has pointed out that RLX-RXFP1 signalling is able to interact (or potentially undergo crosstalk) with other receptors to mediate the therapeutic effects of RLX, including the AT_2_R [27,28,33], Notch-1 [32], glucocorticoid receptor [43,44] and PPAR_Υ_ [45,46]. Furthermore, the results obtained from the in vitro studies conducted confirmed that RXFP1-AT_2_R crosstalk played a central role in facilitating the therapeutic effects of RLX [27,28,33], which now appear to include its ability to inhibit myofibroblast NLRP3 inflammasome activity (Figure 7).

What this study has additionally provided is the novel finding that RLX appears to also modulate the P2X7R on HCMFs and, within the LV of ISO-induced mice with cardiomyopathy, to inhibit NLRP3 inflammasome activity. This is based on the demonstration that the inhibitory effects of RLX (via RXFP1) on measures of NLRP3 inflammasome priming and activity were annulled by the pharmacological blockade of P2X7R (using A-438079; Figure 3, Figure 4, Figure 5 and Figure 6). These results were further confirmed using a selective P2X7R agonist (Bz-ATP) in combination with A-438079 in HCMFs (Figure 2). At the in vitro level, a dose of the P2X7R antagonist (0.05 μM; from the dose-response studies conducted) was used, which did not affect the T+L+Bz- (or T+L+A)-induced increase in myofibroblast NLRP3 inflammasome measures on its own. At the in vivo level, a previously-used dose of A-438079 (34.2 mg/kg/day [47]) was used that had demonstrated the cardioprotective effects of P2X7R blockade in preclinical models of CVD [48,49]. In either case, the co-administration of A-438079 and RLX resulted in the blockade of the cardioprotective effects of RLX in HCMFs and the murine model of cardiomyopathy established. This allows for the inferences (i) that activation of the P2X7R contributed to the detrimental effects of the myofibroblast NLRP3 inflammasome on fibrosis progression, and (ii) that RLX was able to disrupt this P2X7R-mediated activation of the myofibroblast NLRP3 inflammasome as part of its anti-fibrotic actions. Collectively, the findings from this study and previous studies evaluating the signal transduction mechanisms by which RLX targets the myofibroblast NLRP3 inflammasome [34,35] now suggest that RLX acts at multiple levels upstream of the myofibroblast NLRP3 inflammasome, including RXFP1, the AT_2_R and P2X7R, TLR-4 and ROS, and within the inflammasome (at the level of caspase-1), to disrupt the pro-fibrotic contribution of the myofibroblast NLRP3 inflammasome to cardiac fibrosis (Figure 7).

Interestingly, the effects of RLX on DHE-staining-associated superoxide and TGF-β1 expression levels did not appear to be mediated via a P2X7R-dependent mechanism (Figure 6), since the DHE-staining- or TGF-β1-inhibitory effects of RLX were not significantly affected by A-438079 co-administration. These findings may have suggested that the ROS-inhibitory effects of RLX, which were blocked by NAC co-administration, may have more likely involved its ability to restore glutathione levels [50] and/or inhibit the detrimental effects of hydrogen peroxide or other oxidative stress-related metabolites [51,52], rather than regulate superoxide levels. Furthermore, the lack of a marked effect of A-438079 on the RLX-induced down-regulation of TGF-β1 expression levels may have suggested that the crosstalk between RXFP1 and the P2X7R may have more likely occurred at the cellular level (i.e., on macrophages and/or myofibroblasts that express both receptors and the NLRP3 inflammasome), rather than on the cytokines produced by these cells. Further work is required to verify these speculations.

The RLX-induced blunting of LV cardiomyocyte hypertrophy that was observed in this study is somewhat consistent with previous findings demonstrating that RLX (0.5 mg/kg/day) administration alone over a 7 day period did not have a marked impact on LV cardiomyocyte size in ISO-injured mice [37], but significantly reduced LV cardiomyocyte hypertrophy in a murine model of diabetic cardiomyopathy, when continuously administered at the same dose over a 14-day period [53] in vivo. Interestingly, whilst the antagonism of P2X7R activity alone (with A-438079) also only blunted LV cardiomyocyte size after 7 days of treatment in ISO-injured mice, 16 weeks of A438079 (10 or 20 mg/kg/day) administration to mice with diabetic cardiomyopathy prevented the disease-induced increase in the HW:BW ratio and measures of hypertrophic gene expression [54]. These findings suggested that the therapeutic impact of RLX or P2X7R blockade on LV cardiomyocyte size may have been related to the length of treatment exposure and/or the aetiology of disease-induced LV hypertrophy. Strikingly, however, the combined effects of RLX and A-438079 treatment further increased cardiomyocyte size to a higher extent than ISO administration alone or following RLX or A-438079 treatment of ISO-injured mice. This unexpected result may possibly be ascribed to the activation of protein kinase A (PKA) by RLX, which causes an increase in calcium influx within cardiomyocytes due to the prolongation of action potentials [55]. On the other hand, the activation of the P2X7R leads to potassium efflux [6]. Therefore, the co-administration of RLX and A-438079 may have led to an ion imbalance within the cells, maintaining them in a depolarized state, which in turn may have further increased calcium influx. The overload of calcium induces detrimental effects within the cell, which may have triggered an abnormal increase in cardiomyocyte size, although this needs to be confirmed in future studies.

Although the findings of this study are the first to demonstrate that H2 RLX mediates several of its cardioprotective effects via a P2X7R-dependent mechanism, previous studies have curiously shown that H3 relaxin inhibited collagen synthesis via P2X7R-mediated inflammasome activity, when administered to high glucose-stimulated rat cardiac (myo)fibroblasts [56]. Whilst H3 relaxin primarily signals through its own cognate receptor, relaxin family peptide 3 (RXFP3) [57], studies have shown that H3 relaxin can also signal through RXFP1 to exert it anti-fibrotic actions [58], suggesting that the myofibroblast NLRP3 inflammasome-inhibitory effects RLX or H3 relaxin are likely mediated via an RXFP1- and a P2X7R-dependent mechanism.

In conclusion, this study has confirmed that RLX inhibits the myofibroblast NLRP3 inflammasome via a RXFP1-, AT_2_R- and P2X7R-mediated mechanism, and by disrupting the induction of inflammasome at the level of TLR-4, ROS and caspase-1, as part of its anti-fibrotic effects. As such, this is the first study to demonstrate that RLX can mediate its inhibitory effects on myofibroblast NLRP3 inflammasome priming and activity, via the P2X7R. Additionally, it was found that the anti-inflammatory (involving macrophage infiltration and MCP-1 expression levels), anti-hypertrophic (involving LV cardiomyocyte size) and anti-fibrotic (involving α-SMA-associated interstitial myofibroblast differentiation and interstitial LV collagen deposition) effects of RLX appeared to involve the P2X7R, since these therapeutic effects of RLX were blunted or significantly ameliorated by P2X7R antagonism. These findings warrant further investigation of these recently unravelled interactions between RLX-RXFP1 and the P2X7R, to determine: (i) the signal transduction mechanisms by which RXFP1 undergoes crosstalk with the P2X7; (ii) whether this receptor crosstalk only occurs in myofibroblasts or other NLRP3 inflammasome-expressing cells as well; and (iii) whether the crosstalk between RXFP1 and the P2X7R also requires the presence of the AT_2_R (which has been shown to be required for RLX to induce its anti-fibrotic effects [27,28,33], or can occur independently of the AT_2_R. Furthermore, the extent to which the effects of RLX on cardiac function are mediated via the P2X7R needs to be determined. Addressing these questions in additional studies will lead to a better understanding of the receptor crosstalk that is utilized by RLX to induce its pleiotropic effects as an effective anti-fibrotic therapy.

## 4. Materials and Methods

### 4.1. Materials

Primary human cardiac fibroblasts (HCFs; containing a mixture of atrial and ventricular fibroblasts) were purchased from ScienCell (#6300; Carlsbad, CA, USA). Recombinant human TGF-β1 was purchased from In Vitro Technologies (#240-B, Minneapolis, MN, USA). Serelaxin (recombinant human gene-2 (H2) relaxin) was generously provided by Corthera Inc. (San Mateo, CA, USA; a subsidiary of Novartis International AG, Basel, Switzerland). Isoproterenol (ISO; #5984-95-2), LPS (#L2630), ATP (#A6419), Bz-ATP (#B6396), NAC (#A9165), A-438079 (#A9736) and PD123319 (#P186) were purchased from Sigma-Aldrich (North Ryde, NSW, Australia). TAK-242 (#13871) and Ac-YVAD-CHO (#10016) were purchased from Cayman Chemicals (Ann Arbor, MI, USA). B-R13/17K H2 (RXFP1 inhibitor) was chemically produced by Professor Akhter Hossain (Florey Institute of Neuroscience and Mental Health, The University of Melbourne, Parkville, Victoria, Australia).

### 4.2. Culture and Treatment of Human Cardiac Myofibroblasts (HCMFs)

HCMFs were cultured in DMEM supplemented with 1% fibroblast growth factor-2 (FGF-2; ScienCell), 10% foetal bovine serum, penicillin (50 U/mL)/streptomycin (50 μg/mL) and 1% L-glutamine. These cells were passaged when 80–90% confluent and used between passages 2–4 for all outlined studies.

Primary HCFs, which express RXFP1 [34,59], were seeded at a density of 1.25–1.5 × 10^5^/well in 12 well-plates and stimulated with TGF-β1 (T, 5 ng/mL) to differentiate these cells into myofibroblasts (HCMFs). Sub-groups of T-stimulated HCMFs were also treated with LPS (L, 100 ng/mL) and ATP (5 mM) (T+L+A) for 8 h (when NLRP3 inflammasome priming and activity has been shown to be optimally measured [14,34]). To determine whether RLX modulated NLRP3 inflammasome activity via the P2X7R, the dose-response effects of the P2X7R-specific agonist, Bz-ATP (Bz; at 0.1 or 0.2 mM) in T+L+Bz-stimulated HCMFs (seeded at the same density detailed above) were first conducted, to determine the dose of the agonist that promoted NLRP3 (as a measure of NLRP3 priming) and pro-IL-1β (as a surrogate measure of NLRP3 inflammasome activity, based on our previous findings which demonstrated that a T+L+A-stimulated increase in pro-IL-1β from HCMFs correlated with increased IL-1β activity after 8 h [34]), to a similar extent as T+L+A (5 mM). T alone-stimulated HCMFs were included as controls. These studies were conducted *n* = 4 separate times in duplicate. In separate experiments, sub-groups of T+L+A (5 mM)- or T+L+Bz (0.1 mM)-stimulated HCMFs were either left untreated or treated with RLX (RLX or R; 16.8 nM; equivalent to 100 ng/mL) for 8 h, as detailed below.

### 4.3. Determination of the Mechanisms by Which RLX Mediates Its NLRP3 Inflammasome-Inhibitory Actions in HCMFs

To delineate the signal transduction mechanisms by which RLX mediated its NLRP3 inflammasome-inhibitory actions, dose-response studies of a number of pharmacological inhibitors or antagonists to P2X7R (using A-438079 (A4); from 0.05–0.5 µM), ROS (using N-acetylcysteine (NAC); from 1–10 µM), or caspase-1 (using Ac-YVAD-CHO (YVAD); from 0.05–0.5 µM) were evaluated over 8 h in culture in order to obtain a dose of each inhibitor/antagonist that did not affect the T+L+A-stimulated increase in NLRP3, caspase-1 (in the case of YVAD) or IL-1β on their own. These dose-response studies were conducted *n* = 3–5 separate times in duplicate. On the other hand, previously-used doses of the RXFP1 inhibitor (B-R13/17K H2 (RXFP1-I); 1 µM [60,61]), AT_2_R antagonist (PD123319 (PD); 0.1 µM [27,33]) and TLR-4 inhibitor (TAK-242 (TAK); 0.05 µM [34]) that had been shown to antagonize the therapeutic effects of RLX, in the absence of inducing any effects alone, were chosen to be used in subsequent studies.

To determine the effects of RLX at the level of the RXFP1, AT_2_R or P2X7R, sub-groups of T+L+Bz-stimulated HCMFs were treated with or without RLX (T+L+Bz+RLX) and with or without the RXFP1-I (1 µM), or AT_2_R antagonist (PD; 0.1 µM) or P2X7R antagonist (A4; 0.05 µM) for 8 h. These studies were conducted 6 separate times in duplicate, and changes in the protein expression levels of TLR-4, NLRP3, pro-caspase-1, pro-IL-1β and pro-IL-18 were then detected. As our previous studies had determined that functional receptor crosstalk could occur between RXFP1 and the AT_2_R [27,33], co-localization of RXFP1 and P2X7 receptors or AT_2_ and P2X7 receptors was determined using immunofluorescence staining of T+L+Bz-stimulated HCMFs.

To then determine how RLX signalled down-stream of RXFP1, the AT_2_R and P2X7R, separate subgroups of T+L+A-stimulated HCMFs were treated with or without RLX (T+L+A+RLX) and with or without the P2X7R antagonist (A4; 0.05 µM), or TLR-4 inhibitor (TAK; 0.05 µM), ROS inhibitor (NAC; 1 µM), or caspase-1 inhibitor (YVAD; 0.05 µM) for 8 h. These studies were conducted 6 separate times in duplicate, and changes in TLR-4 (for the effects of TAK only), NLRP3, pro-caspase-1 (instead of NLRP3 for the effects of YVAD only), pro-IL-1β and pro-IL-18 were then detected.

### 4.4. ISO-Induced Murine Model of Cardiomyopathy

The 14–16-week-old male 129_sv/ev_ mice used in the study (provided by the Animal Resource Centre, Perth, Western Australia, Australia) were allowed to acclimatize for at least 6-to-7 days prior to experimentation and were maintained on a 12 h light /12 h dark cycle with free access to water and standard rodent chow (Brastoc Stockfeeds, Pakenham, VIC, Australia). All experiments described below were approved by a Monash University Animal Ethics Committee (MARP/2017/034), which adheres to the Guidelines for the Care and Use of Laboratory Animals for Scientific Purposes (National Health and Medical Research Council of Australia, Canberra, ACT, Australia). Initially, groups of equal size were designed using randomization and blinded analysis; however, due to the limited availability of 129_sv/ev_ mice at the time of the study, power calculations were performed to ensure that adequate group sizes were used for the studies detailed below, where it was determined that with a 25% standard deviation, we would be 80% powered to detect a 25–30% effect with *n* = 6–7 animals per group.

To determine if the NLRP3-inhibitory and anti-fibrotic effects of RLX were mediated via the P2X7R in vivo, sub-groups of male 129_sv/ev_ mice were subcutaneously administered with ISO (25 mg/kg body weight) once daily for 5 consecutive days [34,36,37] and then were left for a further 9 days for left ventricular (LV) fibrosis to develop. Thereafter, mice were randomly assigned to the following groups and were either left untreated (ISO-injured control group; *n* = 7) or were treated at day 7 post-injury with either RLX alone (0.5 mg/kg/day, a dose previously used to demonstrate its therapeutic actions in ISO-injured mice in vivo [34,36,37] via s.c-implanted model 1007D osmotic mini-pumps; Alzet, Cupertino, CA, USA; *n* = 6), A-438079 (A4) alone (34.2 mg/kg/day, a dose used previously to demonstrate its P2X7R-inhibitory actions in vivo [47] via daily s.c-injections for 7 days; *n* = 6), or both RLX (0.5 mg/kg/day)+A4 (34.2 mg/kg/day) combined (*n* = 6). All treatments were maintained for a 7-day period, until day 14 post-injury. A separate sub-group of mice given five daily s.c-injections of saline instead of ISO (*n* = 7) and left until day 14 served as the non-injury control group.

### 4.5. Blood Pressure Measurements

Systolic blood pressure (SBP) was determined from each animal, from the 5 groups detailed above, using tail cuff plethysmography (MC4000 Blood Pressure Analysis System; Hatteras Instruments Inc., Cary, NC, USA). The SBP measurements were obtained before the mice were subjected to ISO- or saline-injections (on day 0), prior to any treatment (on day 7) and upon completion of the experimental period (on day 14). At least 15–20 measurements per time point were pooled to obtain a mean value for each animal.

### 4.6. Tissue Collection

On day 14 after the first saline or ISO injection, all mice were weighed and then killed through an anaesthetic overdose (isoflurane; 5% in oxygen), before the heart was isolated from each animal. Heart tissues were weighed, before the LV was separated from the right ventricle and atria and weighed again. The LV from each animal was then separated transversely into three portions, the apex, mid-zone and base. The LV mid-zone from each mouse was placed into separate cryomolds filled with an optimal cutting temperature (OCT) compound (Scigen Scientific, Gardena, CA, USA) and then frozen-fixed before being stored at −80 °C until required. The apex and base from each mouse were separately snap-frozen in liquid nitrogen before being stored at −80 °C until required.

### 4.7. Western Blotting

After 8 h, the cell layer from all HCMF-treated groups was collected following a 10 min incubation period with accutase solution (Sigma-Aldrich, Castle Hill, New South Wales, Australia) at 37 °C. Protein was extracted from the cell layer or from the basal portion of LV tissue from all groups of mice detailed above, using the RIPA lysis buffer (Cell Signaling Technology, Danvers, MA, USA), which was subsequently stored at −20 °C until required. Equivalent volumes of protein samples from HCMF-cell layers or LV tissues (duplicate samples per experiment per gel) were electrophoresed in separate mini-protean 4–15% precast gels and analysed with Western blotting using primary monoclonal antibodies to TLR-4 (95 kDa; AF1478; 1:1000 dilution; In Vitro Technologies, Noble Park North, Victoria, Australia), NLRP3 (110 kDa; #15101; 1:1000 dilution; Cell Signaling Technology, Danvers, MA, USA), caspase-1 (which detected a 42 kDa inactive product and 20 kDa active product; #3866; 1:1000 dilution; Cell Signaling Technology), pro-IL-1β (31 kDa; MAB601-100; 1:1000 dilution; In Vitro Technologies) or pro-IL-18 (24 kDa; D043-3; 1:1000 dilution; In Vitro Technologies). The equivalent loading of protein between samples was confirmed using a monoclonal GAPDH house-keeping antibody (36 kDa; ab8245; 1:1000 dilution; Abcam Australia, Melbourne, VIC, Australia). Membranes were first probed with GAPDH (to ensure equivalent loading of protein), then washed, reblocked and reprobed with 1–2 separate antibodies (ensuring there was no overlap in expected product detection) to detect the various proteins of interest as detailed previously [34]. Each membrane was then subjected to an anti-mouse (#7076) HRP secondary antibody (1:2000 dilution; Cell Signaling Technology) before proteins were detected using the Clarity Western ECL substrate detection kit and quantified using densitometry with a ChemiDoc MP Imaging System and Image Lab v.6.0 software (both from Bio-Rad Laboratories, Hercules, CA, USA). The density of each parameter measured was corrected for GAPDH protein levels and then expressed relative to the TGF-β1 (T) alone-treated group, which was expressed as 1 in each case. Representative blots of the appropriate end points determined were also chosen for presentation in each case.

### 4.8. Histopathology and Immunofluorescence Staining

As the crosstalk between RXFP1 and the AT_2_R had already been established in rat renal myofibroblasts [27] and HCMFs [33], the colocalization of the P2X7 receptor with either RXFP1 or the AT_2_R in HCMFs was performed via double-labelling of RXFP1 and P2X7R or AT_2_R and P2X7R, in T+L+Bz+R-stimulated HCMFs using immunofluorescence staining. HCMFs grown in coverslips were rehydrated, unmasked and blocked in 5% BSA. Coverslips were then incubated overnight at 4 °C with the following antibody pairs: a rabbit polyclonal anti-RXFP1 (#A 9227.1; 1:1000 dilution; Immunodiagnostik AG; Bensheim, Germany) and rat monoclonal anti-P2X7R (ab195356; 1:500 dilution; Abcam, Cambridge, MA, USA) or rabbit polyclonal anti-AT_2_R (#PA5-20813; 1:500 dilution; ThermoFisher Scientific; Scoresby, VIC, Australia) and rat monoclonal anti-P2X7R (ab195356; 1:500 dilution; Abcam). The following day, coverslips were incubated with donkey anti-rat Alexa Fluor 488 (A-11006; 1:500 dilution; ThermoFisher Scientific) and donkey anti-rabbit Alexa Fluor 594 (A-11012; 1:500 dilution; ThermoFisher Scientific) for 2 h at room temperature prior to rehydration in PBS. HCMFs were then mounted using VECTASHIELD HardSet™ Mounting Medium containing DAPI (#H-1500; Vector Laboratories, Meadowbrook, QLD, Australia) to preserve fluorescence. Images were captured using an Olympus BX51 microscope with the appropriate fluorescent filters. Images of HCMFs were sampled at ×200 magnification and then combined using the Image J software (NIH, Bethesda, MD, USA).

Serial frozen mouse LV mid-zone sections (5 µm thickness) from saline- and ISO-injured/treated mice were placed on charged Mikro Glass slides (Grale Scientific, Ringwood, VIC, Australia) and subjected to picrosirius red-staining (0.05%; Polysciences Inc., Warrington, PA, USA). All stained slides were scanned by Monash Histology Services using ScanScope AT (Aperio, Sausalito, CA, USA). Images from the whole tissue section were captured and analysed using the ImageScope software (Aperio). Picrosirius red-stained staining results were expressed as % whole section of interstitial collagen staining. LV cardiomyocyte hypertrophy from at least 100 cells per section were also measured from the picrosirius red-staining sections from each animal (at ×400 magnification).

The effects of RLX (0.5 mg/kg/day) ± A4 (34.2 mg/kg) on measures of LV inflammation and remodelling were determined through immunofluorescence staining of serial OCT-embedded mouse LV mid-zone sections. Sections were rehydrated, unmasked and blocked in 10% normal goat serum, then separately stained overnight at 4 °C with either a mouse monoclonal-monocyte chemoattractant (MCP)-1 (#sc-32771; 1:500 dilution; Santa Cruz Biotechnology, Santa Cruz, CA, USA), rat anti-mouse F4/80 (as a marker of macrophage infiltration; #MCA497R; 1:100 dilution; Bio-Rad Laboratories, Hercules, CA, USA), rabbit polyclonal anti-TGF-β1 (#ab92486; 1:250 dilution; Abcam) or mouse monoclonal anti-α-SMA (as a marker of myofibroblast differentiation; #ab8245; 1:250 dilution; Abcam) primary antibody. The following day, LV sections were incubated with either a goat anti-rat Alexa 488 (#A11006; ThermoFisher Scientific; to detect F4/80), goat anti-rabbit Alexa 488 (#A11008; ThermoFisher Scientific; to detect TGF-β1) or goat anti-rabbit Alexa 594 (#A11012; ThermoFisher Scientific; to detect MCP-1 or α-SMA) secondary antibody for 2 h at room temperature prior to rehydration in PBS. The sections were then mounted using DAPI-containing mounting media (VECTASHIELD HardSet™ Mounting Medium containing DAPI; Abacus DX, Waterford, Queensland, Australia) to preserve fluorescence. Images were captured using an Olympus BX51 microscope with the appropriate fluorescent filters. Images of each section were sampled at ×40–400 magnification and then combined using the Image J software (NIH, Bethesda, MD, USA). In each case, images from 6–8 non-overlapping fields were captured (at ×400 magnification) and analysed, and the data were expressed as the percentage of the fractional area of each end point stained [37].

### 4.9. Determination of LV Superoxide Levels

Superoxide levels present within the LV were determined through dihydroethidium (DHE)-staining, as detailed previously [37,62]. LV tissue sections were air-dried at room temperature for 10 min and then washed with ice-cold 0.01 M PBS for 5 min to remove OCT compound. Afterwards, tissue sections were incubated in PBS or PBS containing 1000 unit/mL of polyethylene glycol superoxide dismutase (PEG-SOD; Sigma-Aldrich; negative control) for 30 min at 37 °C. Following this, DHE (Sigma-Aldrich) was added to each section (containing PBS or PBS+PEG-SOD) to achieve a final concentration of 2 µM and then incubated for 45 min at 37 °C. Ultimately, each tissue section was washed with distilled H_2_O for 2 min to terminate the DHE reaction, then mounted with VECTASHIELD HardSet™ Mounting Medium containing DAPI, and coverslipped. Stained sections were imaged and then analysed utilizing the ImageJ software (v1.8.0; Java, NIH).

### 4.10. Statistical Analysis

All data are expressed as the mean or relative mean ± SEM and were statistically analysed using GraphPad Prism v8.0 (GraphPad Software Inc., San Diego, CA, USA). All data which were not normalised to a house-keeping control were analysed via a one-way ANOVA and Tukey’s post-hoc test to allow for multiple comparisons between groups. All data that were normalised to a house-keeping control and then to a control group (expressed as 1 in each case) were analysed using a non-parametric Kruskal–Wallis test. In each case, data were considered significant with a *p*-value less than 0.05.

## Figures and Tables

**Table 1:** The effects of ISO as well as RLX and/or A4 treatment on systolic blood pressure (SBP), animal body weight (BW), heart and LV weight 14 days post-injury.

Treatment Groups	BW (g)	HW (mg)	LVW (mg)	HW/BW (mg/g)	LVW/BW (mg/g)	d0 SBP (mmHg)	d7 SBP (mmHg)	d14 SBP (mmHg)
Saline (*n* = 7)	26 ± 1	150 ± 4	99 ± 3	5.8 ± 0.1	3.8 ± 0.2	120 ± 5	117 ± 6	119 ± 4
ISO (*n* = 7)	27 ± 1	150 ± 3	105 ± 4	5.6 ± 0.2	3.9 ± 0.1	119 ± 4	118 ± 4	117 ± 3
ISO+A4 (*n* = 6)	28 ± 1	160 ± 6	120 ± 5 *	5.6 ± 0.2	4.2 ± 0.1	123 ± 4	119 ± 4	122 ± 3
ISO+R (*n* = 6)	27 ± 1	143 ± 3	111 ± 4	5.3 ± 0.1	4.1 ± 0.1	116 ± 4	114 ± 4	116 ± 3
ISO+A4+R (*n* = 6)	27 ± 1	147 ± 3	110 ± 3	5.5 ± 0.1	4.1 ± 0.1	117 ± 6	114 ± 4	116 ± 3

Data are presented as the mean ± SEM, with numbers in parentheses representing the number of animals analysed per treatment group. * *p* < 0.05 compared to the saline group.

## Data Availability

The datasets generated and analysed during the study have been maintained within the Monash University data storage, and are available from the corresponding author upon reasonable request.
